# Emerging Tick-Borne *Dabie bandavirus*: Virology, Epidemiology, and Prevention

**DOI:** 10.3390/microorganisms11092309

**Published:** 2023-09-13

**Authors:** Eun-Ha Kim, Su-Jin Park

**Affiliations:** 1 Center for Study of Emerging and Re-Emerging Viruses, Korea Virus Research Institute, Institute for Basic Science (IBS), Daejeon 34126, Republic of Korea; dealereh@ibs.re.kr; 2 Division of Life Science, Research Institute of Life Science, Gyeongsang National University, Jinju 52828, Republic of Korea

**Keywords:** severe fever with thrombocytopenia syndrome, *Dabie bandavirus*, public health

## Abstract

Severe Fever with Thrombocytopenia Syndrome (SFTS), caused by *Dabie bandavirus* (SFTSV), is an emerging infectious disease first identified in China. Since its discovery, infections have spread throughout East Asian countries primarily through tick bites but also via transmission between animals and humans. The expanding range of ticks, the primary vectors for SFTSV, combined with migration patterns of tick-carrying birds, sets the stage for the global spread of this virus. SFTSV rapidly evolves due to continuous mutation and reassortment; currently, no approved vaccines or antiviral drugs are available. Thus, the threat this virus poses to global health is unmistakable. This review consolidates the most recent research on SFTSV, including its molecular characteristics, transmission pathways through ticks and other animals, as well as the progress in antiviral drug and vaccine development, encompassing animal models and clinical trials.

## 1. Introduction

Tick-borne viral diseases are caused by transmitting infectious agents through tick bites [1,2]. Among these viruses, *Dabie bandavirus* (SFTSV, formerly severe fever with thrombocytopenia syndrome (SFTS) virus), belonging to the *bandavirus* genus of the *phenuiviridae* family, was identified in 2009 in China [3]. Subsequently, Korea and Japan reported cases of SFTSV infection in 2013 [4,5]. With the disease considered endemic in China, Korea, and Japan, more than 14,500 SFTS patients were reported by the end of 2019 [6,7,8]. The primary clinical symptoms of severe SFTSV infection include hemorrhagic fever, thrombocytopenia, leukocytopenia, and elevated liver enzymes, which can potentially lead to fatal outcomes [3,9,10,11]. Fatalities mainly occur in patients over 50, with reported mortality rates from 10–19% [7,8,12,13,14,15].

The prevalence of SFTSV is closely related to the ecology of ticks [16,17]. Recently, environmental changes have caused a marked expansion of tick populations and habitats, drawing extra attention to SFTSV as an emerging pathogen of concern [18,19]. While SFTSV has been reported in several East Asian countries, including China, Korea, Japan, Vietnam, and Pakistan, its emergence is threatened in other countries due to the migration of ticks [3,4,5,20,21]. Currently, no treatment is available, and governments recommend that individuals living in rural or forested areas take precautions to avoid tick bites [22]. Therefore, the World Health Organization (WHO) has included SFTSV in its priority target pathogens requiring urgent attention [23].

While SFTSV infects a wide range of animal species in nature, establishing an appropriate animal model for a study depends on whether disease progression aligns with that of human disease [24,25,26,27,28]. However, SFTSV infection is not lethal in most animals, which is a barrier to developing vaccines and antiviral therapies [29,30,31,32,33]. Nevertheless, recent studies have identified animals highly susceptible to SFTSV infection that mimic some SFTS human pathogenesis. Thus, attempts are being made to develop vaccines and antiviral drugs against SFTSV infection in these model systems. This review addresses the current development status against SFTSV to provide a deeper understanding of this virus and the efforts to combat and eradicate this infectious disease.

## 2. Virus Genome and Function

The SFTSV genome is a segmented single-stranded RNA in the genus *Bandavirus*, family *phenuviridae* [34]. The viral genomes are composed of large (L), medium (M), and small (S) genes [3,35]. The L and M segments utilize a negative-sense coding strategy. In contrast, the S segment adopts an ambisense coding strategy for synthesizing nucleoprotein (N), and the nonstructural NSs are separated by a non-coding intergenic region (IGR).

The L segment encodes the RNA-dependent RNA polymerase (RdRp) and has an N terminal endonuclease domain, polymerase core, and C terminal cap-binding domain [36,37]. The L protein initiates viral transcription through cap snatching, where the viral RdRp protein binds to the 5′ cap of the host mRNA using its cap-binding domain, cleaves the RNA using its endonuclease domain, and elongates the RNA [36,37,38,39,40]. Furthermore, genome replication by RdRp is initiated de novo, and full-length viral RNA is synthesized using the complementary antigenomic RNA (cRNA) intermediate [41].

The M segment encodes the Gn and Gc envelope glycoproteins derived from polyprotein precursors, major components on the viral surface that target specific neutralizing antibodies [42]. For viral entry and cell penetration, these proteins bind the cellular receptors such as dendritic cell-specific intercellular adhesion molecule-3-grabbing non-integrin (DC-SIGN), expressed on macrophages and dendritic cells [43,44]. Moreover, lymph node sinusoidal endothelial cell C-type lectin (LSECtin), as well as glucosylceramide synthase (UGCG) and non-muscle myosin heavy chain IIA (NMMHC-IIA), have also been involved in cell attachment and entry [45,46,47]. During virus entry and penetration, the Gc proteins exhibit fusion activity triggered to transport virions into endolysosomes around pH 5.6–6 and release the RNP complex composed with vRNA, RNA-dependent RNA polymerase (RdRp), and N proteins [46,48].

The S gene encodes both the N and NSs proteins. The mRNA of the N is transcribed from the viral sense, and NSs is transcribed from the antisense RNA intermediate. Upon association with the RdRp, the N protein interacts with viral RNA, and this complex functions as the RNA synthesis machinery (RNP) in the cytoplasm [49,50,51,52]. The RNP complex assembles translated Gn and Gc in the ER-Golgi intermediate compartment (ERGIC) or Golgi apparatus from which infectious virions then exit [52,53]. The NSs protein has been found to function in evasion of the innate immune response [54,55,56,57]. Upon viral infection, the retinoic acid-inducible gene I (RIG-I), responsible for recognizing viral RNA, is ubiquitinated by the tripartite motif-containing protein 25 (TRIM25). The subsequent activation of RIG-I and MDA5 facilitates the initiation of the antiviral response through their interaction with the mitochondrial antiviral-signaling protein (MAVS) [58,59,60]. Once activated, MAVS recruits the TBK1 and IKKε signaling molecules to the complex, allowing the recruitment of the activated interferon regulatory factor (IRF) 3 transcription factor and subsequent interferon (IFN) production [61,62]. To circumvent this, NSs has the unique ability to form cytoplasmic granules, in which it sequesters several host proteins and factors, including TRIM25 and TBK1 [55,63,64]. As a result, RIG-1 and IRF3 demonstrate reduced activation, resulting in restricted induction of IFNs [63]. Virus infection also alters the IFN-mediated response through STAT2 [65,66]. The binding of secreted IFN to its receptors activates the IFN signaling pathway through phosphorylation and activation of receptor-associated kinases TYK2 and JAK1, which subsequently phosphorylate the STAT1 and STAT2 transcription factors [67]. Phosphorylated STAT1 and STAT2 dimerize and associate with IRF-9 to form the interferon-stimulated gene factor 3 (ISGF3) complex, which translocates into the nucleus and activates antiviral IFN-stimulated genes (ISGs) [68]. The viral NSs protein can bind directly to STAT2 and sequester it into NSs granules, disrupting type I IFN signaling in the cytoplasm [66]. Therefore, NSs is a significant virulence factor that potently hinders the innate immune response of the host.

## 3. SFTSV Genotype

### 3.1. Pure Genotype

Previous phylogenetic analysis classified SFTSV strains into Chinese (C1–C5) and Japanese (J1–J3) clades, indicating separate evolution and genetic diversity of SFTSV strains from different locations [69,70]. However, isolated viral strains subsequently demonstrated the inconsistency of geographic distributions. Fu et al. analyzed SFTSV strains isolated from China, Korea, and Japan and classified six genotypes as A–F. This classification was calculated based on genotype mean distances [71]. The Chinese clade was further subdivided into genotypes A, C, D, E, and F, dominant in China, while the Japanese clade was renamed as genotype B and has mainly circulated in Korea and Japan. Following phylogenetic tree analysis, genotype B strains were subdivided into three different subgenotypes, B-1, B-2, and B-3, with strong bootstrap values (>70) [14]. Moreover, recent findings suggest that all clades of viruses are present in China, and A, B, C, D, and F clades have been found in Korea and Japan [14,72]. Therefore, the increased incidence of SFTSV suggests that an A–F genotype classification is more suitable, even though recent genotype classifications have typically used the Chinese/Japanese clade and the A–F clade.

### 3.2. Reassortant Genotype

Viral evolution can occur by reassorting genome segments between strains co-infecting the same host [73]. Vectors that are suitable hosts for coinfection increase the chances of genome reassortment and can spread viruses [74]. Hence, the co-circulation of viruses and their migration among different geographical locations, possibly facilitated by birds, are significant contributors to the emergence of new virus strains through reassortment [75]. Consequently, phylogenetic analyses of the L, M, and S segments suggest that approximately 6–11% of SFTSV strains are classified as reassorted viruses [14,72,76].

Given that reassortment events have been previously reported among *Bunyaviridae* viruses, there are growing concerns about the potential for reassortment events to occur via their RNA segments [77,78,79]. One example of such a reassortant virus is *Ngari orthobunyavirus*, which resulted from the exchange of RNA segments between *Bunyamwera orthobunyavirus* (BUNV) (donor of S and L segments) and *Batai orthobunyavirus* (BATV) (donor of the M segment) [77]. Rezelj et al. demonstrated the possibility of reassortment events among *Uukuniemi phlebovirus* (UUKV), *Heartland bandavirus* (HRTV), and SFTSV using a minigenome system [80]. Their findings revealed that the SFTSV N and RdRp proteins could utilize UUKV and HRTV M UTRs in the minigenome assay. These findings indicate that the viruses have the potential to generate novel reassortant viruses.

## 4. The Potential Primary Vector for the Spread of SFTSV

Although the transmission cycle and origin of SFTSV are not fully understood, sever-al tick species have been found to carry SFTSV, including *Rhipicephalus microplus*, *Rhip-icephalus sanguinensis*, *Haemaphysalis concinna*, *Haemaphysalis longicornis*, and *Haemaphysa-lis flava* [16,81,82]. Studies suggest that the dominant hard-tick species in China, Korea, and Japan, *Haemaphysalis longicornis*, might be one of the primary vectors of SFTSV. Moreover, this tick species reproduces sexually and parthenogenetically [83,84,85]. Parthenogenesis, a type of asexual reproduction, allows female ticks to establish new populations without embryo fertilization, resulting in a more rapid population expansion than ticks utilizing sexual reproduction [19,86,87]. Further, the high proportion of parthenogenetic ticks found on migratory birds captured in the SFTSV endemic area was found to correlate with the distribution of parthenogenetic populations and SFTS cases in China, suggesting that these birds could be responsible for the long-distance movement of ticks in China. The distribution of *Haemaphysalis longicornis* aligns with the occurrence of SFTSV along the East Asia/Australasia flyway, a route used by migratory birds such as the White’s Thrush, Grey-backed Thrush, Ruddy Kingfisher, Fairy Pitta, and Naumann’s Thrush [19,87]. A recent study also revealed that Swan Geese and Spotted Doves carry antibodies against SFTSV [17].

The spread of parthenogenetic ticks has also been observed in the Eastern United States, where they were first identified in New Jersey in 2017 and have since been found in 18 states, primarily in the East [88,89,90]. This rapid expansion of ticks in this region may be linked to migratory bird species like the Gray Catbird, Blue Jay, and House Wren [91].

Moreover, resident birds could also contribute to the dissemination of SFTSV by harboring *Haemaphysalis longicornis*. Notably, antibodies against SFTSV have been identified in several bird species in East Asia, including rock pigeons, pheasant pigeons, and turtle doves [92]. Furthermore, ticks were discovered on various resident bird species, such as the Carolina Wren, Canada Goose, chicken, Brown Booby, and Northern Cardinal in the Eastern United States [91,93]. Additionally, with global warming, there may be an expansion or shift in the distribution of *Haemaphysalis longicornis* populations [93,94,95,96]. Thus, SFTSV is likely spread by *Haemaphysalis longicornis*, and the increased distribution of these ticks increases the possibility of SFTSV emergence.

## 5. Virus Transmission

### 5.1. SFTSV Maintenance and Transmission by Ticks

*Haemaphysalis longicornis* thrives in a broad temperature spectrum, ranging from −2 to 40 °C, and is found in grassy and forested areas [97,98]. These ticks undergo four life stages: egg, larva, nymph, and adult [96,99]. Except for the egg stage, each stage of ticks must feed on blood before they molt into the next stage or lay eggs, in the case of adult females. During feeding time, ticks can transmit viruses to various animals, including poultry, livestock, wild animals, and humans [100]. Although humans are not the primary hosts of SFTSV and are regarded as incidental hosts, they are susceptible to infection when bitten by infected ticks [101,102]. Notably, individuals involved in agriculture or that spend extended periods in grassy or wooded areas are at increased risk [103,104]. Consequently, a higher prevalence of SFTSV infections has been reported in rural areas than in urban environments. Moreover, the prevalence of this virus in rural areas also suggests an elevated risk of infection for companion animals.

Furthermore, the maintenance of the virus is not limited to tick bites. The virus can be vertically transmitted from a female parent tick directly to its offspring or horizontally, from an infected tick to a host, or vice versa, from an infected animal to a tick. In experiments infecting ticks, SFTSV was found to disseminate to the ovaries and salivary glands, suggesting infected ticks can transmit the virus vertically and horizontally [83,105]. The detection of SFTSV in saliva and hemolymph implies that the virus is circulating within the body cavity and is secreted in saliva. However, even though the coxal gland is active during apolysis in ixodid ticks, there is no evidence of transmission through coxal secretion [106,107]. Compared to the persistence of viruses in other tick species, the genetic sequence of SFTSV in adult *Haemaphysalis longicornis* ticks can maintained for up to 21 days. This is significantly longer than in other species: 18 days in *Inimicus sinensis*, 9 days in *Ixodes persalcatus*, and 6 days in *Dermacentor silvarum*. This longer carrying time is essential for transmitting SFTSV [16].

### 5.2. Virus Transmission by Infected Animals or Humans

While ticks are believed to be the primary vectors for the transmission of SFTSV mainly through their bite, the virus can also be transmitted by infected animals or humans [9,108,109,110,111,112,113]. Studies indicate that infected individuals can transmit the virus to family members or healthcare workers while hospitalized, mainly through contact with infected blood or body fluids [9,10,110,111,114]. The virus is present in tracheal and gastric aspirates, urine, and other mucosal secretions of patients, which may lead to transmission or result in asymptomatic cases [115,116]. Notably, there was a report of an SFTSV-infected patient transmitting the virus to family members even while they wore gloves and masks for protection [117]. This report suggests a possible risk of infection through the eyes, underscoring the need for healthcare workers to adopt additional protective equipment such as goggles or face shields. Furthermore, the proper segregation and disposal of virus-contaminated biomedical waste and body secretions is needed to prevent secondary infections [118].

Transmission of SFTSV from infected companion animals, such as cats and dogs, to humans has also been reported through various routes [109,111,114,119,120]. The virus can be transmitted to owners through direct contact with the body fluids of infected animals, and bites by infected animals [119]. There have also been cases where humans were infected without any direct contact with infected animals, suggesting transmission may also occur through aerosols, as demonstrated in instances where veterinary personnel contracted SFTSV despite wearing gloves and surgical masks and not being bitten or scratched by a hospitalized cat [109,111,114]. In these cases, the virus was detected in the serum of the infected personnel, and isolated virus sequences showed 100% homology with that isolated from the cats [112,113].

In an experimental study, four of six ferrets co-housed with infected ferrets demonstrated virus infection from two to ten days after contact [121]. The infected and transmitted ferrets exhibited clinical symptoms such as elevated body temperature and weight loss. Additionally, the virus was detected in body fluids, such as blood, stool, nasal wash, saliva, and urine of inoculated and direct-contact ferrets. Therefore, this study suggests that SFTSV-infected hosts can transmit the virus through various body secretions, potentially causing lethal infections in close contact.

## 6. Diagnosis

The clinical manifestations of early stage SFTS can be non-specific, asymptomatic, or similar to symptoms like scrub typhus and anaplasmosis [116,122,123,124]. Therefore, accurate diagnosis is crucial for providing appropriate care, controlling viral transmission, and effectively managing the disease.

Researchers have developed numerous methods to detect viral RNA [125,126,127,128,129,130]. The prevalent approach targets the highly conserved regions of the L, M, or S genes across various genotypes, facilitating detection in suspected samples from humans and animals. Among these detection methods, real-time PCR (RT-PCR) is the gold standard in terms of accuracy and sensitivity [125,126]. However, recent advancements have led to the emergence of diagnostic tools like reverse transcription-loop-mediated isothermal amplification (RT-LAMP) and CRISPR-based nucleic acid detection [127,128,129,130]. These methods offer rapid detection times of under an hour and sensitivity levels comparable to RT-PCR.

Enzyme-linked immunosorbent assays (ELISA) and immunofluorescence assays (IFA) are commonly used for serological diagnosis. However, these techniques exhibit a lower sensitivity when compared to RNA detection [131,132]. According to a study, within the first 7 days following disease onset, RT-PCR identified 52.2% of positive samples [132]. Conversely, only 19.7% tested positive for the SFTSV-specific IgM antibody within the same time frame. IgM antibodies typically reach peak detection between 8–14 days post-onset, while IgG antibodies peak around 1.5 months. While serology titers can assist in diagnosing viral infections, serological tests, particularly those detecting IgG, are valuable for contact tracing and assessing herd immunity.

## 7. Lethal Animal Model for SFTSV

### 7.1. Mice and Hamster

Although genetic variations between mice and humans can result in diverse disease outcomes after pathogen infections, mice are still considered a primary animal model due to their small size, ease of handling, experimental reproducibility, and low cost [133,134]. Following identifying SFTSV, researchers aimed to identify a suitable mouse model to study virus characteristics, pathogenicity, host interaction, and drug/vaccine efficiency. Mice aged between 6 and 12 weeks and those older than one year from strains including C57BL/6, CH3, FVB, BALB/c, and ICR exhibit limited viral replication and disease progression [31,33,135]. To induce lethality in mice infected with SFTSV, Jin et al. administered mitomycin C to C57BL/6 mice at a dose of 0.02 mg daily for 3 days before infection, followed by a reduced daily dose of 0.001 mg for 3 days after infection. Half of these mice succumbed after being infected with 10^5^ TCID_50_ of HB29 SFTSV [31] (Table 1). Chen et al. also demonstrated that mitomycin C-treated pregnant mice can transmit the virus to the fetus through the placental barrier, causing fetal damage [136]. The newborn mice are lethal but unsuitable for demonstrating the efficacy of drugs and vaccines [30,32]. Therefore, immunocompetent strains of mice are an insufficient model system for investigating human SFTSV infections.

Similar to SFTSV, several other viruses, including HRTV, Zika, and West Nile virus, replicate poorly and fail to cause disease in standard mouse strains [137,138,139]. To investigate the pathogenesis of these viruses as well as the efficacy of vaccines and antiviral therapies, mice lacking functional genes related to the immune system are utilized. Among immune-deficient mice, type I IFN-deficient (IFNAR^−/−^) mice are highly susceptible to SFTSV infection and exhibit lethality [32,140,141,142]. Although there are slight variations in platelet levels among SFTSV-infected IFNAR^−/−^, infected mice developed similar clinical manifestations such as weight loss, ruffled fur, and depression with viral replication in multiple organs and excreta. Histopathological analysis of spleens revealed histiocytic and necrotizing splenitis, loss of white pulp, and diffuse red pulp. The liver showed coagulation necrosis and mononuclear inflammatory cell infiltration, and the destruction of lymphoid follicles was also observed in the cervical lymph nodes. Additionally, the susceptibility of mice lacking STAT2 to SFTSV infection is comparable to that of IFNAR^−/−^ mice, a similarity also observed in the golden Syrian hamster STAT2 knockout model [143,144].

Although several mouse models demonstrated lethal outcomes of SFTSV infection, hemorrhagic symptoms were limited. To overcome this, Xu et al. developed a humanized mouse model by engrafting NCG mice with human PBMCs [145]. This model allowed the virus to have an early replicating target, the human PBMCs, and infected human monocytes can transmit the virus to murine monocytes in a cell–cell manner. As a result, the pathogenic mechanisms of hemorrhage syndrome were elucidated into apoptosis, membrane protein endocytosis, and cytokine stimulation. This model showed that viral replication occurred not only in the spleen and liver but also in the brain and intestine. Hematological changes, including AST, ALT, CK, thrombocytopenia, and leukocytopenia, were also observed, along with increased levels of inflammatory cytokines in the blood, similar to what is seen in human SFTS cases. However, while humanized NCG mice are ideal for assessing effector and memory T-cell and NK-cell function, further studies are needed to demonstrate the immune response concerning B cells [146].

**Table 1:** Lethal animal model against SFTSV.

Animal Model	Summary of Key Findings	References
C57BL/6 with mitomycin C treatment	loss of body weight	[31]
	viral transmission from pregnant to the fetus	[136]
Newborn		
C57BL/6, BALB/c, Kunming	necrotic region within the liver of the Kunming	[30]
CD-1	tremors, loss of balance	[32]
IFNAR^−/−^	weight loss, ruffled fur, depression	[32,140,141,142]
	virus replication in multiple organs	
STAT2^−/−^	weight loss	[143,144]
	virus replication in multiple organs	
	changes in hematology	
	changes in serum biochemistry parameters	
Humanized mouse (human PBMC into NCG mice)	weight loss	[145]
	virus replication in multiple organs	
	changes in hematology	
Aged ferret	weight loss, body temperature increase	[135]
	virus replication in multiple organs	
	changes in hematology	
	changes in serum biochemistry parameters	
Cat	weight loss, body temperature increase	[147]
	gastrointestinal symptom	
	increase in proinflammatory cytokine and chemokine	
	virus replication in multiple organs	

### 7.2. Age-Dependent Ferret

Ferrets are relatively small and have anatomical and physiological similarities to humans [148]. Since ferrets (*Mustela putorius furo*) were identified as a susceptible animal model for the influenza virus, they have been widely used to study various viruses, including SARS-CoV, SARS-CoV-2, rabies virus, and Nipah virus, where they exhibit clinical symptoms similar to those observed during human infections [149,150,151,152,153,154]. Park et al. have shown that SFTSV infection in ferrets is age-dependent, with aged ferrets (≥4 years of age) being more susceptible than young adults (≤2 years of age) (Table 1) [135]. Infected, aged ferrets demonstrated high fever, weight loss, and hematological changes, including thrombocytopenia, leukopenia, and increases in AST/ALT levels, similar to severe human infection cases. Furthermore, the virus replicated in multiple tissues, including the brain, spinal cord, lung, spleen, liver, kidney, intestine, and serum, and animals succumbed to death within 10 days post-infection. In contrast, young adult ferrets demonstrated fewer clinical symptoms, with only 5% weight loss, mild temperature changes, and hematological changes returning to normal levels within 16 days post-infection. Transcriptome analysis of aged-ferret PBMCs also revealed that IFN and IRF signaling pathways effectively suppress early virus infection and proliferation, leading to rapid clearance of SFTSV from young adult ferrets. However, in aged ferrets, SFTSV infection triggers delayed IFN and IRF responses alongside persistently upregulated inflammatory immune responses. This persistent inflammation leads to tissue damage and contributes to increased mortality.

Infected aged ferrets also showed virus transmission to contact animals and demonstrated virus secretion in excreta, indicating the possibility of SFTSV infection in animal models [93]. Despite their compatibility, the use of ferrets in studies is limited by factors such as genetic heterogeneity (out-bred), the requirement of additional facilities, a lack of knowledge about their immunological system, and a paucity of reagents [150,155].

### 7.3. Cat

Cases of SFTSV infection in cats have been reported in Korea and Japan [156,157]. SFTSV-infected cats transmit the virus to humans through aerosol transmission, infected blood, or other bodily fluids [112,113,157]. Park et al. demonstrated that 66% of cats intravenously infected with SFTSV succumbed within 10 days of infection (Table 1) [147]. Moreover, the infected cats exhibited clinical signs such as fever, weight loss, gastrointestinal symptoms, leukocytopenia, and severe thrombocytopenia, which resemble severe clinical manifestations of human infections. Proinflammatory cytokine and chemokine levels were also elevated, and histological analysis showed hematolysis in the spleen, lymph node, and bone marrow. The virus was detected in multiple organs of cats, including the liver, spleen, kidney, brain, and body fluids, suggesting that direct contact with infected animals could lead to virus transmission. Therefore, SFTSV infection in cats is lethal, and a feline model should be considered for SFTSV infection studies.

## 8. SFTSV Vaccine

### 8.1. Inactivated Vaccine

Vaccines are a cost-effective and efficient method of controlling infectious diseases. Traditionally, inactivated vaccines, such as those for hepatitis A and influenza, have been used and are known to be safe and effective at preventing diseases [158,159]. These vaccines are created by propagating pathogens in a culture medium and then inactivating them using chemicals like ß-propiolactone, formaldehyde, or detergents [160,161].

Li et al. examined the response to vaccination with a whole SFTSV virion, inactivated using ß-propiolactone, at doses of 0.25, 2, and 8 μg, with or without aluminum hydroxide adjuvant, in BALB/c and C57BL/6 mice [160]. Mice were vaccinated three times, two weeks apart. All vaccinated mice demonstrated SFTSV-specific IgG and neutralizing antibodies two weeks after the last vaccination. Thus, this indicates that the inactivated virus particles induced humoral immunity in C57BL/6 mice. Moreover, no virus was detected in serum from mice vaccinated with the intermediate or high dose following the challenge with SFTSV. In contrast, the virus was detected in the challenged, low-dose-vaccinated mice. These results indicate that the inactivated vaccine is a promising candidate for preventing SFTSV infection.

### 8.2. DNA Vaccine

DNA vaccines are easy to design, stable, and have a low cost of production. Recent clinical studies have shown that they effectively prevent infectious diseases, such as those caused by HIV-1, Zika virus, Ebola virus, MERS-CoV, and influenza viruses [162]. Two DNA vaccine candidates against SFTSV were investigated in IFNAR^−/−^ mice and aged ferrets.

In a study by Kang and colleagues, the Gn, Gc, NP, and NSs genes were incorporated into a vector (pSFTSV) [163]. Additionally, they included the IL-12 gene in pSFTSV (pSFTSV-IL-12), to enhance cell-mediated immunity. These plasmids were subsequently immunized to IFNAR^−/−^ mice by in vivo electroporation three times at two-week intervals. After the final immunization, only the mice vaccinated with pSFTSV-IL-12 demonstrated detectable levels of NP antibodies, whereas Gn and Gc were barely detectable. However, the pSFTSV-IL-12 group exhibited Gn and NP-specific CD4^+^ and CD8^+^ T cell responses compared to the vector group. Two weeks after the final immunization, all immunized mice were challenged. The group of mice transfected with pSFTSV-IL-12 demonstrated 100% survival, while the group transfected with pSFTSV showed only 40% survival. Meanwhile, the vector-alone group did not survive the challenge. This study demonstrated that although immunization with Gn, Gc, NP, and NS genes without IL-12 genes is not fully protective against infection, DNA vaccines provide some degree of protection against SFTSV infection in IFNAR^−/−^ mice.

Kwak et al. manufactured plasmids individually encoding full-length Gn, Gc, N, NSs, or the RdRp genes [164]. Aged ferrets were immunized with a mixture of all five SFTSV DNA plasmids (Gn, Gc, N, NSs, and RdRp) or vector three times at 2-week intervals. Animals immunized with SFTSV genes elicited SFTSV-specific T cell responses and exhibited neutralization titers of more than 80. Two or four weeks after the last vaccination, immunized ferrets were challenged with a lethal dose of SFTSV. The SFTSV gene-immunized groups showed no clinical signs of infection, a normal range of hematological changes, and only low-level virus titers were detected on day two post-challenge. In contrast, vector-immunized animals demonstrated SFTS syndrome and died. This study suggests the potential of DNA vaccines as an effective strategy for preventing SFTSV.

### 8.3. Viral Vector Vaccine

Viral vectors are a promising tool for delivering genes that produce desired antigens and are commonly used in vaccine development to combat infectious viruses. Consequently, many viral vectors are evaluated for efficacy as potential candidates for protection against SFTSV infection.

Dong et al. developed a recombinant Vesicular stomatitis virus (rVSV)-expressing SFTSV Gn/Gc, referred to as rVSV-SFTSV/AH12-GP, as a viral vector vaccine [165]. The rVSV-SFTSV/AH12-GP demonstrated decreased virus replication in VeroE6 cells and decreased pathogenicity in IFNAR^−/−^ mice compared to the original rVSV-G (rVSV). To investigate the efficacy of rVSV-SFTSV/AH12-GP as a vaccine, IFNAR^−/−^ mice were immunized with it, and their sera were collected 30 days after immunization. The serum from these immunized mice exhibited a neutralization titer of 4 to 8 against SFTSV. The immunized mice survived with a 1000LD_50_ SFTSV challenge, indicating that the rVSV platform vaccine is a promising candidate for developing an SFTSV vaccine.

The Vaccinia virus (VAC) strain LC16m8 (m8) has previously been used as a live recombinant vaccine for smallpox in humans [166,167,168]. Yoshikawa et al. generated m8-based SFTSV vaccine candidates that express the SFTSV N protein (m8-N), glycoprotein precursor (m8-GPC), or both N and GPC (m8-N + GPC) and evaluated their efficiency in IFNAR^−/−^ mice [169]. The mice were subcutaneously inoculated twice with each m8-based SFTSV vaccine at a 2-week interval. After the last immunization, serum was collected, the IgG antibody titers against the glycoprotein precursor or N protein were between 210 and 213. After challenge with either 1 × 10^3^ or 1 × 10^5^ TCID_50_ of SFTSV YG-1 SFTSV, the m8-N + GPC and m8-GPC groups showed protection, while the m8-N group was less protected, with death occurring after high-dose challenge. Although the authors could not elucidate the mechanism by which N antibodies reduce viral replication in this study, the m8-based SFTSV vaccine was effective at inducing protection against SFTSV.

Zhao et al. demonstrated that a bivalent vaccine can hinder the infection of both SFTSV and rabies virus [170]. A replication-deficient recombinant human adenovirus type 5 (Ad5), co-expressing either rabies virus G and SFTSV Gn (Ad5-G-Gn) or GFP (Ad5-GFP), was manufactured. C57BL/6 mice were immunized with a single dose of Ad5-G-Gn, Ad5-GFP, or mock vector at 10^8^ GFU. After 4 weeks of immunization, the mice receiving the Ad5-G-Gn virus showed significantly higher antibody titers against rabies virus and SFTSV than the Ad5-GFP or mock-immunized groups. Specifically, the Ad5-G-Gn immunized mice exhibited a fluorescent antibody virus neutralization titer of 47.57 IU/mL against rabies virus and 102 FRNT_50_ against SFTSV. The Ad5-G-Gn immunized group showed no clinical signs after being challenged with rabies virus, whereas the other groups succumbed to death. Furthermore, when challenged with SFTSV, mice in the Ad5-G-Gn group exhibited decreased viral titer compared to the other groups. Therefore, the Ad5-G-Gn vaccine targeting rabies and SFTSV is efficient in mice.

These findings suggest that viral vectors have significant potential for developing effective vaccines against SFTSV.

### 8.4. Live Attenuated Vaccine

Live attenuated vaccines consist of live viruses that do not cause severe disease, as they are engineered to have decreased replication ability in the body. Yu et al. studied the effectiveness of two attenuated mutant viruses as potential vaccine candidates against SFTSV [171]. These mutants had a single mutation in NSs, proline to alanine at position 102 (rHB29P102A), and NS deletion, with only 12 amino acids derived from the NSs proteins (rHB2912aaNSs), in the rHB29 backbone. Aged ferrets infected with these mutant viruses did not exhibit clinical signs, such as weight loss or temperature changes, unlike those infected with rHB29, which showed slightly increased fever and weight loss. None of the ferrets infected with the mutant viruses died, while those infected with Korean-isolated genotype D (the same genotype as rHB29) exhibited more than 10% weight loss, increased body temperature, and finally succumbed to 100% mortality. The neutralization titers of the ferrets infected with the mutant viruses were more than 2.5 × 10log_2_ FRNT_50_ after 58 days of infection. After 58 days of infection, ferrets infected with the rHB29, rHB29NSsP102A, or rHB2912aaNSs viruses survived a lethal challenge with the B genotype virus, while non-immunized ferrets succumbed within 10 days after the challenge.

Furthermore, immunized ferrets showed no clinical signs of infection, such as weight loss or temperature changes, and maintained a normal range of hematological parameters. Given its diminished pathogenicity and ability to mimic natural infection, this live attenuated vaccine shows promise as a countermeasure against SFTSV. This live attenuated vaccine demonstrates a promising defense against SFTSV due to its reduced pathogenicity and ability to mimic natural infection. Collectively, these findings emphasize the effectiveness of modified NSs-containing live attenuated vaccines for preventing SFTSV, further validating their appropriateness for developing vaccine strategies.

## 9. Therapeutics

### 9.1. Antiviral Drugs Efficiency In Vivo

Ribavirin and favipiravir (T-705), nucleoside analogues that inhibit the synthesis of viral RNA, are promising antiviral drugs against RNA viruses [172,173]. Tani et al. demonstrated the efficacy of these drugs in vitro, with the 50% inhibitory concentrations (EC_50_) of ribavirin and T-705 being 6.0 µM and 49 µM in Vero cells, respectively [140]. To evaluate efficacy in vivo, IFNAR^−/−^ mice infected with a lethal dose of SFTSV were treated with 25 or 100 mg/kg of ribavirin and 60 or 300 mg/kg of T-705 for 5 days, respectively, through intraperitoneal or oral administration. Mice treated with ribavirin showed more than 20% weight loss and 40% mortality. However, mice treated with T-705 showed 100% survival rates with less than 10% body weight loss, and viral clearance was observed within 7 days of infection. Therefore, T-705 is a more promising antiviral drug against SFTSV than ribavirin.

During FDA-approved antiviral screening, Li et al. identified that benidipine hydrochloride, a calcium channel blocker (CCB), inhibited virus binding, internalization, and fusion of the virus envelope with the endosomal membrane in Vero cells against SFTSV [174]. Several other CCBs, including benidipine hydrochloride, also exhibited inhibition of SFTSV replication, leading to further evaluation in vivo. SFTSV infected NOD. Cg-Prkdc^em1IDMO^Il2rg^em2IDMO^, treated with these drugs, showed reduced viral titers in their serum and spleen. All nifedipine-treated mice survived, while benidipine hydrochloride-treated mice had only an 83.7% survival rate. In comparison, non-treated mice had a survival rate of 57.1%. Consequently, nifedipine is considered a promising antiviral candidate for treating SFTSV infections.

The antiviral activity of 2′-deoxy-2′-fluorocytidine (2′-FdC), also known as 2′-fluoro-2′-deoxycytidine (2′-FdC), has been demonstrated against a wide range of viruses [175,176,177]. In a study using SFTSV-infected IFNAR^−/−^ mice, 2′-FdC was administered and compared with T-705-treated and non-treated groups [175]. Mice treated with 2′-FdC showed continuous weight loss, similar to those observed in non-treated mice; however, they recovered after 10 dpi, showing a survival rate of over 80%. In contrast, mice treated with T-705 did not lose weight and exhibited 90% survival, while the non-treated mice were all dead. Further, the 2′-FdC- and T-705-treated groups showed significantly reduced viral titers compared with the non-treated mice. These results suggest that T-705 is a more effective antiviral drug than 2′-FdC against SFTSV infection.

### 9.2. Antiviral Drug Efficiency in Clinical Data

As ribavirin demonstrated inhibition of viral replication in vitro, it was initially approved by the Chinese Ministry of Health to treat SFTS [178]. Thus, after identifying SFTS in patients, they were treated with ribavirin, although its effectiveness against SFTS remained controversial [179,180,181,182,183]. A meta-data analysis by Chen et al. and Liu et al. found no association between the administration of ribavirin and survival of SFTS [180,183]. Nevertheless, ribavirin therapy was found to increase the survival rate in patients with a low viral load (<1 × 10^6^ copies/mL) upon admission but not in those with high viral loads, compared to those who were not given ribavirin, despite no change in virus titers [181]. Zhang et al. conducted a study to evaluate the effectiveness of ribavirin treatment (600 mg qd in adults, and 200 mg qd in children) in SFTSV patients [183]. The ribavirin treatment groups did not show significant changes compared to the non-treated group. However, patients who received ribavirin within 4 days of symptom onset demonstrated a significantly reduced duration of viral titer compared to those who received ribavirin after 5 days of symptom onset or who were not treated.

Li et al. conducted clinical trials to investigate the efficacy and safety of T-705 in SFTS patients [184]. The drug-treated patients were allocated randomly and received 1800 mg twice on the first day and 1000 mg twice on day 2 and continuing for at least 5 days or until the detection limit of viral copy number was reached or the patient was discharged. Patients who received T-705 exhibited a shorter time to viral clearance (5.6 ± 2.1 vs. 6.8 ± 2.8 days), as well as reduced hemorrhagic signs and rapid recovery of neutrophils and lymphocytes. However, there was no significant difference in survival rates between the patients who received the drug and those who did not. The authors also categorized the T-705-treated patients into low and high viral load groups upon admission. T-705 treatment in the low viral load group resulted in a rapid decrease in viral load and a reduced case fatality rate (1.6%) compared to the non-treated group (11.55%) with reduced hemorrhagic signs and neurological symptoms.

In contrast, the group with a high viral load showed no significant effectiveness of T-705 treatment in terms of survival probability (60% vs. 46.2%), even though viral clearance was faster than that of the non-treated group. Moreover, to identify the mutagenesis mechanism of T-705, mutation analyses were conducted on 74 serum samples collected from 12 T-705-treated patients and 11 control patients. Increased numbers of viral mutations were seen in surviving patients and not in fatal cases after treatment with T-705. Thus, T-705 appears to potentially induce clearance of SFTSV by terminating viral replication through mutation.

Nifedipine, a calcium channel blocker, also underwent clinical trials as it was also found to inhibit viral replication both in vitro and in a mouse model [174]. The clinical trial consisted of three groups: the nifedipine-treated group (administered nifedipine before and after admission), the non-nifedipine-treated group (given nifedipine before admission but ceased after), and the general group (not treated with nifedipine). The nifedipine-treated group initially had high viral titers (≥10^6^ copies/mL), but had a significantly lower fatality rate compared to the other groups (2.4% vs. 29% and 34.5%, respectively). Furthermore, a group of nifedipine-treated patients also exhibited a reduced case fatality rate overall with a reduction in neurological symptoms, such as coma and lethargy, as well as hemoptysis, in comparison to the other groups; however, other clinical symptoms, including platelet counts and neutrophil percentages, were similar.

These findings suggest that while Ribavirin, T-705, and nifedipine show some potential in managing SFTSV, additional extensive clinical trials are needed to determine their effectiveness, time for treatment, and efficiency, depending on the dosage.

## 10. Conclusions

Due to the increasing incidence of SFTSV infections in Asia, the World Health Organization prioritized research and development of SFTSV treatments and therapeutics by classifying it as an emerging disease in 2018. Furthermore, warming due to climate change has raised concerns about the potential for an increase in the habitat of SFTSV-carrying ticks in areas where the emergence of the virus was previously unknown. Along with this, vertical transmission from parents to eggs of tick species could result in the continuous spread of the virus. As a result, suitable animal models have been identified to facilitate the efficient and safe approval of vaccines and drugs for clinical application against various genotypes and recombinant SFTSV.

While there are currently no FDA-approved antiviral drugs for SFTSV, numerous clinical trials are underway. For example, nifedipine, when administered promptly, has been associated with improved survival rates. This is consistent with findings in patients over 65 with SARS-CoV-2 infections; those with milder symptoms demonstrated significant improvements with early antiviral treatment [185]. Moreover, no adverse effects were noted for patients treated with T-705 or nifedipine, although these results are based on a single trial. Conversely, ribavirin treatment led to potential side effects such as anemia and hyperamylasemia. Therefore, a thorough assessment of factors, including potential side effects and dosage, is essential when considering antiviral treatments. In addition, advancements in human research may lead to adapting these treatments for use in companion animals infected with SFTSV. Currently, companion animals are only given supportive care in veterinary hospitals, which includes antimicrobial and symptomatic treatments [186,187].

Currently, vaccine development for SFTSV has exclusively consisted of animal studies, as clinical studies for humans have not yet been conducted. However, clinical studies for SFTSV vaccines will be needed to assess their safety and efficacy, thereby determining their suitability. In addition, vaccination may prove to be a successful approach to preventing animal infections. Given that SFTSV can be transmitted from animals to humans, it may be worthwhile to identify the applicability of these vaccines in companion animals as well as stray cats, and dogs as a means to minimize transmission between animals and humans. These studies will be crucial in determining the effectiveness of vaccines for preventing SFTSV transmission, and both animal and human health.
